# Layilin: a multifunctional hyaluronan receptor in physiology and pathology

**DOI:** 10.3389/fonc.2025.1721065

**Published:** 2025-12-01

**Authors:** Chenchen Jin, Yunfeng Zong

**Affiliations:** 1 Zhejiang Academy of Science & Technology for Inspection & Quarantine, Hangzhou, Zhejiang, China; 2 Department of Pharmacology & Immunology, Medical University of South Carolina, Charleston, SC, United States; 3 Hollings Cancer Center, Medical University of South Carolina, Charleston, SC, United States

**Keywords:** layilin, *LAYN*, extracellular matrix, hyaluronan receptor, therapeutic target

## Abstract

Layilin (encoded by *LAYN*), a C-type lectin transmembrane receptor, serves as a critical molecular bridge between extracellular matrix (ECM) sensing and intracellular signaling through its interaction with cytoskeletal adaptors. Initially identified for its cytoskeletal functions, layilin has since emerged as a pleiotropic modulator of both physiological homeostasis and pathological conditions. Elevated expression of layilin is associated with poor prognosis in multiple cancers, thereby highlighting its oncogenic potential. Beyond cancer, it plays a pivotal role in rheumatoid arthritis, fibrotic progression, and chronic inflammatory diseases. This review comprehensively synthesizes the structural features, expression dynamics, and disease mechanisms of layilin, emphasizing its biological functions. Key knowledge gaps persist, particularly in understanding its spatiotemporal regulation and crosstalk with immune checkpoints. Future research should prioritize cell-type-specific mechanistic studies using advanced experimental models and the development of layilin-targeted immunotherapies. These efforts will pave the way for precise interventions in diseases driven by the dysregulation of layilin-dependent ECM signaling.

## 1 Introduction

Layilin, encoded by the *LAYN* gene, is a transmembrane receptor that was first identified in 1998 (1) for its interactions with cytoskeletal regulators, including talin (2), merlin, and radixin (3). Its unique ability to bridge extracellular matrix (ECM) signals with intracellular responses has positioned it as a critical player in cell adhesion and migration (1). Although early studies have focused on these functions, emerging evidence has revealed its pleiotropic functions in pathological processes, including tumor progression, inflammatory disorders, and immune dysregulation. However, the molecular mechanisms underlying its cell type-specific and context-dependent roles remain poorly understood.

The functional significance of layilin is closely linked to its interaction with hyaluronan (HA) (4), a glycosaminoglycan whose biological effects highly depend on its molecular weight (5, 6). High-molecular-weight HA (HMW-HA, > 500 kDa) maintains tissue homeostasis and promotes anti-inflammatory responses, whereas low-molecular-weight HA (LMW-HA, < 120 kDa) is associated with pro-inflammatory responses (7). Notably, layilin exhibits a distinct preference for binding LMW-HA (< 70 kDa) (8), suggesting that it is a specialized sensor of tissue inflammation and a key mediator of immune responses in the tumor microenvironment (TME) (9). This unique binding specificity underscores the role of layilin in distinguishing between pro-inflammatory and anti-inflammatory signals, making it a critical regulator of tissue homeostasis and disease progression.

This review aims to provide a comprehensive synthesis of the current knowledge on layilin, encompassing its structural features, expression patterns, and dual roles in physiological homeostasis and disease pathogenesis. We particularly focus on its involvement in tumor progression, inflammatory disorders, and signaling networks, highlighting its potential as a cancer immunotherapy and a therapeutic target for inflammatory diseases. By addressing critical gaps in our understanding of the molecular mechanisms and context-dependent functions of layilin, this review seeks to guide future research and therapeutic developments.

## 2 Characteristics of layilin

**Figure 1:**
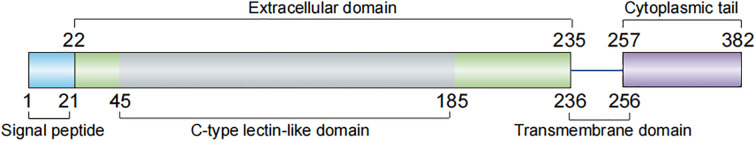
Molecular structure of human layilin. The full-length layilin protein is composed of four distinct domains: a signal peptide (aa 1–21), extracellular domain (aa 22–235), transmembrane domain (aa 236–256), and cytoplasmic tail (aa 257–382). aa, amino acid.

Layilin is a 55 kDa type I transmembrane glycoprotein that serves as a critical molecular link between ECM components and intracellular cytoskeletal networks. Initially identified through its interaction with talin in membrane ruffles (1), its expression has been documented in humans, hamsters, mice, and pigs (1, 10). Layilin is encoded by the *LAYN* gene on human chromosome 11q23.1 (UniProt ID: Q6UX15). The full-length 382-amino acid (aa) protein is composed of four distinct domains (**Figure 1**): a signal peptide (aa 1–21); extracellular domain (aa 22–235), featuring a C-type lectin-like domain (aa 45–185) that explicitly recognizes HA; transmembrane domain (aa 236–256) characterized by LAYILI sequence that gives the protein its name; and cytoplasmic tail (aa 257–382), which contains two functional YXXΦ motifs (YNVI and YDNM) (1) and binding interfaces for both talin’s F3 domain (2) and ERM family proteins (radixin/merlin) (3). These structural features indicate that layilin orchestrates and enables dynamic membrane-cytoskeleton coupling during cell migration and may regulate receptor internalization via clathrin-mediated endocytosis (1). Its expression is particularly prominent in actively migrating cells, where it localizes to leading-edge ruffles, underscoring its role in cell motility and adhesion dynamics (1). An intriguing, unexplored question is whether the considerable energy demands of layilin-mediated migration necessitate an interface with metabolic pathways, akin to the role of MFSD8 in endothelial metabolism (11). Addressing this, along with the precise mechanisms regulating its interactions, remains a crucial goal for future investigation.

## 3 Regulation of layilin expression

Layilin expression is widely and tightly regulated across diverse cell types (1). Its expression is dynamically controlled through cytokine-dependent mechanisms that display remarkable cell-type specificity. In immune cells, T-cell receptor (TCR) activation combined with either interleukin-2 (IL-2) or transforming growth factor-beta (TGF-β) stimulation potently induces the expression of layilin in regulatory T cells (Tregs) (12). In contrast, vascular endothelial growth factor-A (VEGF-A) upregulates layilin in activated CD8^+^ T cells through the transcription factor NR4A1 (13). Inflammatory mediators exert divergent effects on the expression of layilin. Tumor necrosis factor-alpha (TNF-α) upregulates layilin expression in both chondrocytes (14) and human renal carcinoma cells, whereas TGF-β lacks this effect in renal carcinoma cells (15). Similarly, Murata et al. (16) reported that layilin expression in human chondrocytes and synoviocytes was downregulated by interleukin-1 beta (IL-1β), potentially modulating HA-mediated signaling pathways. The pathological context further expands the regulatory spectrum of layilin expression. For example, the community-acquired respiratory distress syndrome (CARDS) toxin induces layilin upregulation in A549 cells (17), suggesting a potential role in host-pathogen interactions. Collectively, these findings establish layilin as a versatile microenvironmental sensor whose expression is precisely controlled by multiple regulatory networks, including immune cytokines and pathogenic factors. This intricate regulation underscores the functional adaptability of layilin in physiological and pathological contexts.

## 4 Ligand recognition and membrane signaling

Layilin functions as a multifunctional receptor that mediates diverse extracellular signals via its ligand-binding capacity. Bono et al. (4) identified HA as a principal ligand, establishing the role of layilin in ECM-cytoskeleton communication, cell motility, adhesion, spreading, and signal transduction. Forteza et al. (8) reported that cigarette smoke-derived LMW-HA binds to layilin in the airway epithelium, activating the RhoA/ROCK signaling pathway. This pathway suppresses E-cadherin expression and increases the epithelial permeability. Conversely, Kim et al. (18) observed that 35-kDa HA (HA35) binding to layilin upregulates zonula occludens-1 (ZO-1) expression, enhancing tight junction integrity and reducing intestinal permeability both *in vitro* and *in vivo*. Bellos et al. (19) further corroborated this protective effect during short-term ethanol exposure. These opposing outcomes highlight the diverse and context-dependent functional roles of layilin signaling networks. The clinical success of HA therapy in inflammatory disorders illustrates the potential of modulating the HA pathway (20). Layilin, given its specificity for pro-inflammatory LMW-HA, thus emerges as a rational target for developing next-generation, receptor-specific anti-inflammatory strategies. Beyond HA, Glasgow et al. (21) identified glycosylated collagens as additional ligands for layilin, underscoring its capacity for immune modulation and its multifunctionality across different biological contexts. Furthermore, the functional spectrum of layilin extends to organelle regulation. Tsutiya et al. (22) revealed its mitochondrial targeting and control over fission dynamics through cyclin-dependent kinase 1 (CDK1) and dynamin-related protein 1 (DRP1) activation, adding mechanistic depth to its pleiotropic functions. Collectively, these findings suggest that layilin is a versatile receptor that integrates extracellular ligands with membrane signaling and organelle homeostasis, highlighting its critical role in diverse physiological and pathological processes.

## 5 Role of layilin in cancer

**Figure 2:**
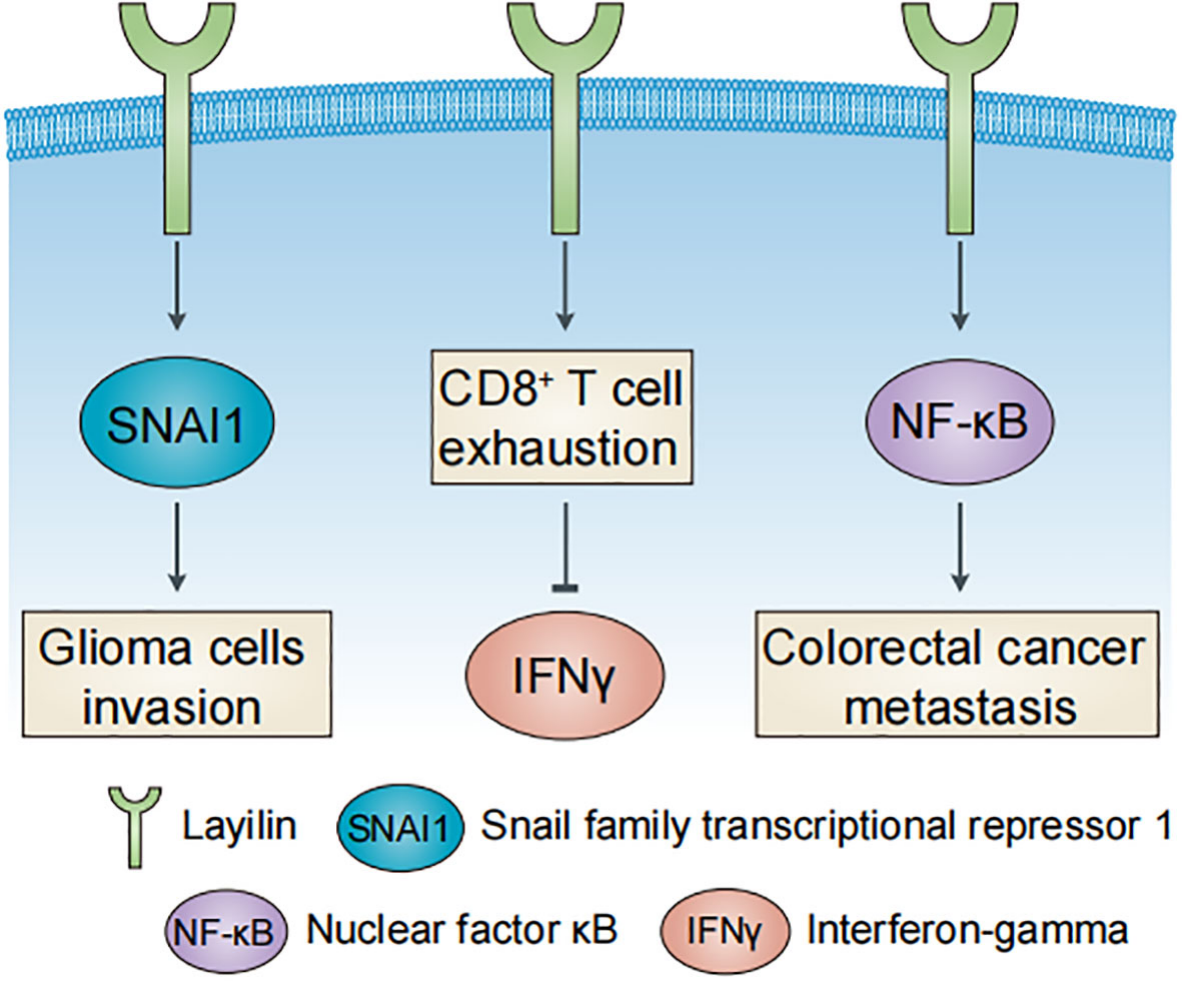
Major roles of layilin in human cancers. Layilin promotes glioma cell invasion, colorectal cancer metastasis, and CD8^+^ T cell exhaustion in liver cancer.

Emerging evidence highlights the multifaceted roles of layilin in oncology (**Figure 2**), influencing both tumor-intrinsic mechanisms and the TME. In tumor cells, layilin promotes malignant glioma invasion through snail family transcriptional repressor 1 (SNAI1), which suppresses nuclear metastasis associated 1 family member 3 (MTA3) (23), while also inhibiting low-density lipoprotein (LDL) uptake (24). Genetic suppression of layilin reduces metastatic progression and extends survival in murine models of lung adenocarcinoma (25). MALAT-1, a long non-coding RNA (lncRNA), mediates *LAYN* upregulation, enhancing tumor cell motility and implicating layilin as a driver of lung cancer aggressiveness (26). This places layilin among the growing list of genes under lncRNA control. This paradigm is further exemplified in other biological contexts, such as by lncRNA Gm2044 during germ cell development (27). Mälarstig et al. (28) identified layilin as a relevant factor in tumor development in breast cancer, while Vogeley et al. (29) demonstrated that layilin facilitates the adhesion of mesenchymal stem cells (MSCs) in breast cancer cells, but not glioma cells, thereby promoting metastatic progression.

Within the TME, layilin drives colorectal cancer metastasis and CCL20 secretion via the NF-κB signaling pathway to recruit tumor-associated macrophages (TAMs) (30). In liver cancer, layilin marks exhausted CD8^+^ T cells that suppress interferon-gamma (IFNγ) production (9). Elevated expression of layilin predicts a better response to anti-VEGFR2 and anti-PD-1 combination therapy in lung adenocarcinoma, which is mechanistically linked to the downregulation of layilin in tumor-infiltrating CD8^+^ T cells (13). However, in melanoma, layilin enhances the cellular adhesiveness and cytotoxic potential of CD8^+^ T cells through integrin αLβ2 (LFA-1) interaction (31). These contrasting findings underscore the context-dependent role of layilin in CD8^+^ T cells and highlight the need for further investigation of its function within the TME.

Bioinformatics studies have consistently associated elevated *LAYN* expression with poor prognosis in multiple malignancies, including hepatocellular carcinoma (9, 32), skeletal undifferentiated pleomorphic sarcoma (33), gastrointestinal cancers (34, 35), breast cancer (28, 36), lung cancer (37, 38), and head and neck squamous cell carcinoma (39). Pan-cancer analyses further link *LAYN* to immunosuppressive features and metastatic potential (40, 41), although the underlying mechanisms remain to be fully elucidated. Collectively, these findings suggest that layilin is a multifunctional regulator of tumor immunity and progression, and its effects are likely dependent on cellular context, tumor type, and TME. The apparent contradictions in the functions of layilin highlight the complexity of its roles and underscore the need for systematic investigations to fully explain its therapeutic potential in cancer settings.

## 6 Layilin and other diseases

Layilin plays diverse roles in inflammatory and fibrotic diseases across multiple organ systems, and pleiotropic roles in tissue homeostasis and disease pathogenesis. In renal diseases, layilin contributes to fibrotic progression by mediating TNFα-induced epithelial-mesenchymal transition (EMT) during glomerulonephritis (15) and serves as a risk biomarker for kidney failure progression (42). Elevated layilin levels in children with Mycoplasma pneumoniae pneumonia (MPP) predict the development of plastic bronchitis (17). Additionally, layilin participates in airway epithelial homeostasis by facilitating apical actin cap formation and multiciliated cell (MCC) differentiation (10). Shimazaki et al. (43) demonstrated that layilin regulates EMT-related proteins in synovial fibroblasts in rheumatic diseases, while Asano et al. (14) identified its involvement in cartilage degradation in joint diseases, such as rheumatoid arthritis and osteoarthritis. Future work should investigate whether layilin contributes to EMT by interfacing with the transcriptional circuits that regulate this process in diverse pathologies (44). Emerging evidence also links layilin to systemic lupus erythematosus (SLE). Altered layilin expression in monocytes has been proposed as a potential contributor to SLE pathogenesis (45). As evidenced by impaired tissue repair in mice with Treg cell-specific *LAYN* deletion, layilin is essential for cutaneous wound healing (12, 46) and skin inflammation (47). Moreover, layilin has been associated with allergic diseases (48) and cellular senescence (49), suggesting its broad involvement in immune dysregulation and aging-related processes. These findings demonstrate that layilin functions as a pleiotropic modulator of tissue inflammation and homeostasis, and its diverse functions are likely dependent on cellular context and disease state. Looking forward, key questions remain regarding the molecular basis of layilin’s pleiotropy. It is crucial to determine if it operates through a core immunometabolic interface, similar to the SIRT6-mediated link between metabolism and inflammatory control (50). Furthermore, the paradigm of single-cell transcriptome-based biomarker prediction (51) offers a powerful strategy to unravel layilin's tissue-specific signaling heterogeneity and accelerate the development of layilin-targeted clinical markers.

## 7 Conclusions

Layilin has emerged as a functionally versatile transmembrane receptor that plays a pivotal role in mediating dynamic crosstalk between ECM signaling and immune regulation. An open question remains whether layilin, much like the interferon-regulated transcriptional control observed in other immune modulators (52), employs a similar mechanism to direct immune cell function. Its unique molecular architecture enables context-dependent modulation of cellular responses, contributing to its dual roles in diseases, including cancer, inflammation, and organ fibrosis. Therapeutically, layilin represents a promising target, not only in combination with immune checkpoint blockade to overcome resistance (13), but also within the broader context of its potential intersections with metabolic and transcriptional networks. However, critical knowledge gaps remain, including its cell type-specific actions, spatiotemporal regulation of ligand-receptor interactions, and crosstalk with canonical immune pathways. Future research should prioritize the development of cell-selective therapeutic strategies to target disease-driving functions while preserving its physiological roles. These advances will accelerate the translation of layilin biology into precision medicine for tumors and inflammatory diseases, potentially establishing new paradigms for treating therapy-resistant conditions (53).
